# Validation of the 10-Item Connor–Davidson Resilience Scale: The Case of Russian Youth

**DOI:** 10.3389/fpsyt.2021.611026

**Published:** 2021-02-10

**Authors:** Sofya Nartova-Bochaver, Aleksei Korneev, Konstantin Bochaver

**Affiliations:** ^1^Department of Psychology, National Research University Higher School of Economics, Moscow, Russia; ^2^Department of Psychology, Lomonosov Moscow State University, Moscow, Russia

**Keywords:** resilience, well-being, validity, reliability, youth, instrumental study

## Abstract

This study validates the 10-item *Connor–Davidson Resilience Scale* (CD-RISC-10) on a Russian youth sample. A total of 689 respondents participated (*M*_*age*_ = 20.22, *SD*_*age*_ = 2.08; 526 females). The *Warwick–Edinburgh Mental Well-being Scale*, the *International Positive and Negative Affect Schedule Short-Form*, the *Centre of Epidemiological Studies-Depression Scale*, the *Rosenberg Self-Esteem Scale*, and the *Authenticity Scale* were used to examine the content validity of CD-RISC-10. Two hypotheses were examined: that the Russian version of the CD-RISC-10 (1) has structural validity (is unifactorial, as the original version) and (2) has convergent validity (which is proven by positive connections with psychological well-being and negative connections with ill-being). According to confirmatory factor analysis (CFA), it was shown that the scale really had a unifactorial structure; its reliability was satisfactory (α =.85, ω_*h*_ =.84). No age trends in the CD-RISC-10 scores were detected; in males, the scores were higher than in females. As expected, CD-RISC-10 was positively connected with mental well-being, positive affect, self-esteem, and authentic living while negatively connected with depressive symptoms, negative affect, acceptance of external influence, and self-alienation. The Russian version of CD-RISC-10 seems to be a valid, stable, and reliable instrument which may be recommended for use in various areas of research and practice.

## Introduction

According to Connor and Davidson (1), resilience is a personal trait which helps people thrive in the face of adversity and bounce back after stressful events, tragedies, or traumas. In today's fast-changing world, it is necessary for everyone to have high resilience to stress. This is especially true for Russia, a country that has undergone several painful political and economic reforms over the past decades, which has affected the mental well-being of its population (2).

Research shows that the most vulnerable ages during the life course are youth and emerging adults (3–6), probably due to the fact that they have to solve many developmental tasks (separation from the parents' family, economic independence, and the choice of education, occupation, and romantic partner) and to cope with the crisis of the first quarter of life. Youth is a very flexible time, full of transitions and uncertainties, so young people need personality traits that help them cope with life's difficulties, first of all—resilience.

Resilience is associated with a variety of related traits, states, and processes that indicate mental and somatic well-being: the absence of trauma experience, posttraumatic stress disorders, emotional distress, and general stress (7–12) and the quality of sleep (13). Moreover, resilience is positively connected with health; positive and higher personal phenomena, such as spirituality (7) and religiosity (14); self-esteem (11, 14); efficient coping strategies (15) and perceived social support (15); and life satisfaction, optimism, positive affect, and general psychological well-being (16, 17). Combined, these traits prove that resilient people are not only better adapted to reality but also more likely to live “proper,” authentic lives.

The lack of resilience is manifested in the inability to respond to life's challenges and temptations, which, in turn, may result in various problems: crimes (18), the first manifestations of schizophrenia and other illnesses (19), and other harmful risk behaviors (20), such as alcohol use and gambling, that occur when people are young. Considering the variety of developmental trajectories in morality (21), we can state that the youth need resilience more than any other age.

To prevent the decrease of mental well-being and to assess the efficiency of the relevant interventions, scholars and practitioners need a stable and reliable diagnostic tool. Diagnostics of resilience has become possible due to a standardized method developed by Connor and Davidson (1). Initially, the *Connor–Davidson Resilience Scale* (CD-RISC) was developed as a self-report scale comprising 25 items and five factors. The structure of the questionnaire was found not stable across social groups and cultures; however, the number of working items was sometimes 21 or 22 only (22–24), and the number of factors varied from one to six (25–29), which damaged possible cross-cultural research.

Many attempts to revise the 25-item CD-RISC (16, 30, 31), to make it shorter and more reliable, have been made. To date, three versions exist: 25-, 10-, and 2-item scales (32). The most stable is the CD-RISC-10 version developed by Campbell-Sills and Stein (33): after removal of 15 items from the initial list of statements, the remaining 10 items were the best at capturing the core features of this phenomenon. CD-RISC-10 has been thoroughly investigated across many samples, cultures, and even continents regarding its reliability, structure, and content validity. Thus, it appears to work well in people of different demographics and occupations as well as in the special samples (7, 8, 13–15, 17, 34–41). As far as we know from literature, the translation of the scale's statements into foreign languages did not have any problems, since they were initially formulated very clearly and did not allow for discrepancies or misunderstandings. All of the researchers followed the recommendations of the World Health Organization (42) and Sousa and Rojjanasrirat (43) regarding the steps of translation (8–11, 42) or, at least, performed forward and back translations. Although not all authors described the translation process in detail in their articles, approval of the translation by the authors of the original scale was a prerequisite for using the questionnaire, which guarantees high quality of translations. To date, there are more than 80 translations of different CD-RISC versions (44). It is also translated and used in Malayalam (45).

In all versions, the unifactorial structure of the scale was preserved (except 14, 40, 41). Cronbach's *alpha* ranged from 0.82 to 0.92, which demonstrates the reliability of the tool. Content validity, examined by correlations with subjective and behavioral indicators of well- or ill-being, was also very convincing (7, 8, 13, 16, 17, 33).

The outcomes describing sex and age differences in the CD-RISC score are scarce and mixed. In some studies, it was found that the mean scores in men were higher than those in women (33, 39).

Although CD-RISC-10 has been becoming increasingly more popular around the world, including in Russia (46), to our best knowledge, the systematic verification of its psychometric properties has not yet been carried out in Russia. The current paper validates CD-RISC-10 for Russia.

Previous research and theorizing allowed us to put forward the following *hypotheses* regarding the psychometric characteristics of the Russian CD-RISC-10 version. Since the scale showed high cross-cultural invariance, we assumed that its structure would be preserved in Russia as well. As the resilience measured by this scale was closely related to other measures of psychological well-being and positive functioning, we expected the tool to have similar connections in Russia.

Hence, the Russian version of CD-RISC-10 has the following:

(H1) has structural validity (is unifactorial, as in the original version); and
(H2) has convergent validity expressed in (a) positive correlation with indicators of well-being (positive affect, mental well-being, self-esteem, and authentic living) and (b) negative correlation with indicators of mental ill-being (negative affect, depressive symptoms, accepting external influence, and self-alienation).

As evidence for age trends was mixed in previous research and because our sample was not representative across all age ranges and was mostly female, we did not put forward special hypotheses regarding differences in CD-RISC-10 by age and sex.

## Methods

### Participants and Procedure

A total of 689 respondents participated in the correlational study (*M*_age_ = 20.22, SD_age_ = 2.08; 526 females M_age_ = 20.25, SD_age_ = 2.17, and 153 males *M*_age_ = 20.13, SD_age_ = 1.76). We used a convenience sampling strategy; data were collected in a series of different research projects, so the sample sizes for different tools varied slightly. All participants were students of Russian universities; data were collected as a part of their homework on “individual differences,” “stress psychology,” and “health psychology” conducted by the authors, during 2017–2020 using 1ka.si (https://www.1ka.si). They were recruited from different levels (499 of them were bachelor students, and 190 of them were master students) and represented humanities and technical specialties. The study was conducted in accordance with the Declaration of Helsinki and approved by the National Research University Higher School of Economics Committee on Interuniversity Surveys and Ethical Assessment of Empirical Research. All of the respondents provided their written informed consent to participate in this study and to publish the results anonymously.

For the translation, the short version^1^ presented in the paper by Campbell-Sills and Stein (33) was chosen. It consisted of 10 items describing components of resilience, such as the ability to adapt to change and to see the humorous side of life. Respondents had to assess the level of their resilience during the last week using a five-point scale, from 0 (not true at all) to 4 (true nearly all the time).

During the translation process, the authors strictly followed the World Health Organization (42) rules and recommendations, which defined the four steps of preparing the questionnaire text.

First of all, the original CD-RISC-10 items were translated separately by two independent Russian researchers; they discussed edited items and came to a consensus. Some wordings (1, 18, 20, 21) were feminized. Secondly, a bilingual expert checked a draft of the questionnaire regarding possible discrepancies in wordings, their clarity, and their accuracy. After this examination, the list of items was again slightly edited. Thirdly, this version was sent to a bilingual Russian psychologist who had been working in a UK university for more than 7 years for back-translation. The statements that were different from the original ones after the back-translation were edited in Russian and re-translated into English. There were several such iterations until the optimal translation was obtained. The final version was approved by Dr. Jonathan Davidson, one of the authors of the original version of CD-RISC. Fourthly, the final version was given to a group of 30 students to check whether the wordings were easily understandable. After this test, no more editing was required.

### Instruments

We used our Russian CD-RISC-10 translation as a main tool, which, in accordance with the purpose of our study, should have been validated in Russia. Five additional measures were included in this study to assess the convergent validity of CD-RISC-10, as they included concepts familiar to or overlapping with resilience and have been already adapted for Russian culture. In addition, when selecting methods, we tried to reproduce the procedure for checking the convergent validity, which was used in some existing adaptations (for instance, 7–12, 14, 17–18).

The *Warwick–Edinburgh Mental Well-being Scale* (WEMWBS) developed by Tennant et al. (47) and adapted in Russia by Nartova-Bochaver (48) is a unidimensional scale measuring respondent self-reported mental well-being during the last 2 weeks. It had 14 items and a five-point scale.

The *International Positive and Negative Affects Schedule Short-Form* (PANAS-SF) has been developed by Thompson (49) and revalidated in Russian by Osin (50), consisted of two subscales describing positive or negative states and emotions, and captured feelings over the last week on a five-point scale.

The *Centre of Epidemiological Studies-Depression Scale* (CES-D) by Radloff (51) was adapted for Russia by Andryushchenko et al. (52). This tool reflected individual self-reported personal states in the past week. It included manifestations of psychosomatic symptoms, interpersonal problems, and proportion of the positive and negative emotions. It consisted of 20 items using a four-point scale.

As resilience and self-esteem are similar constructs, we used the *Rosenberg Self-Esteem Scale* (Self-Est) (53) in the adaptation by Prihozhan (54) measuring an individual's attitude toward the self. There were 10 items with responses on a five-point scale.

Finally, the *Authenticity Scale* by Wood et al. (55) adapted by Bardadymov (56) and Nartova-Bochaver et al. (57) was chosen to measure the convergent validity of CD-RISC-10. It is a three-factor questionnaire measuring three aspects of personal authenticity: *authentic living, accepting external influence*, and *self-alienation*; the last two subscales are reverted. It included 12 items on a seven-point scale.

### Data Analysis

The responses of all participants from different studies were aggregated in one database and were analyzed as one dataset. The reliability was tested by Cronbach's *alpha* (58) and McDonald's *omega* (59). To confirm the unifactorial structure of the Russian version of CD-RISC-10 (H1), we used confirmatory factor analysis (CFA) (maximum-likelihood method). The differences between sex and age groups were estimated by Student's *t*-test, and Cohen's *d* was calculated to measure the effect size. For investigation of correlations between CD-RISC and other indicators of well-/ill-being (H2a and H2b, convergent validity), we used Pearson's correlation.

We used R ver. 4.0.0 (60) for the statistical analysis. Reliability analysis was conducted using psych package ver. 1.9.12.31, and the CFA was conducted using lavaan package ver. 0.6–6 (61).

## Results

### Descriptive Statistics

The descriptive statistics of separated items and the scale is presented in Table 1. The results show that the scores of both separated items and the total averaged scores are negatively skewed (the standard error of skewness is 0.092). The skewness reflects more frequent answers near the upper scale pole. This means the sensitivity of the scale is higher in its lower part.

**Table 1:** Descriptive statistics, reliabilities, factor loadings, and residuals (in parentheses) of the SD-RISC-10 items.

**No**	**Item**	**Mean**	**Standard deviation**	**Skewness**	**α if deleted**	**CFA factor loadings**
1	I am able to adapt to change.	2.962	0.843	−0.815	0.83	0.607 (0.632)
2	I can deal with whatever comes.	2.698	0.848	−0.667	0.825	0.682 (0.535)
3	I try to see the humorous side of problems.	2.425	1.180	−0.311	0.833	0.539 (0.710)
4	Coping with stress can strengthen me.	2.774	1.126	−0.688	0.831	0.568 (0.677)
5	I tend to bounce back after illness or hardship.	2.377	1.122	−0.297	0.824	0.635 (0.597)
6	I can achieve goals despite obstacles.	2.740	0.931	−0.446	0.82	0.724 (0.476)
7	I can stay focused under pressure.	2.007	1.125	−0.088	0.831	0.572 (0.673)
8	I am not easily discouraged by failure.	1.826	1.084	0.178	0.824	0.628 (0.606)
9	I think of myself as a strong person.	2.554	1.144	−0.509	0.82	0.695 (0.517)
10	I can handle unpleasant feelings.	2.418	1.060	−0.351	0.816	0.729 (0.468)
Total		2.478	0.674	−0.169		

### Reliability

We tested the reliability of the scale by Cronbach's *alpha* (58); the coefficient for the CD-RISC-10 is 0.84 with a 95% confidence interval [0.82; 0.86], which is very close to the original version (.85). Also, we calculated McDonald's *omega* (59) to estimate the general factor saturation of the test; the result is similar: ω_*h*_ = 0.84. In addition, we estimated the changes of Cronbach's alpha if any item was deleted. The results (see Table 1) show that reliability decreases if any item was deleted. Therefore, we cannot distinguish any “weak” items in our version of CD-RISC-10.

### Factor Analysis

We used CFA to test the structure of the scale (see Table 1). We used the *WLSMV* estimator, which is robust to non-normally distributed variables and is better for modeling categorical or ordered data (62). The fit indices of the model are good: χ^2^(35) = 131.646, *CFI* = 0.987, *TLI* = 0.983, *RMSEA* = 0.063 [0.052;0.075], *SRMR* = 0.050. The standardized coefficients of the model are presented in the second column of **Table 3**. Hence, the Russian version of CD-RISC-10 has the same factor structure as the original one, confirming Hypothesis 1.

#### Age and Sex

The correlation between the age of the respondents and CD-RISC-10 is not significant (*r* = 0.045, *p* = 0.239).

In Table 2, the results of the comparison of the separated items and the scale in males and females are presented. The analysis reveals statistically significant differences between males and females in items 2, 5, 8, and 10 and in the total score. In all cases, the average result is higher in males than in females, but the effect size according to Cohen's *d* is moderate or low.

**Table 2:** Sex differences in the average item and total scores of CD-RISC-10.

**Item**	**Females (*n* = 526)**	**Males (*n* = 163)**	**CI**	***t*-test**	**Cohen's *d***
1	2.939 (0.812)	3.037 (0.936)	[−0.258; 0.063]	*t*(242) = −1.200, *p* = 0.231	*d* = −0.116 [−0.292; 0.06]
2	2.66 (0.849)	2.822 (0.838)	[−0.311; −0.014]	*t*(273) = −2.155, *p* =0.032	*d* = –.192 [−0.368; −0.016]
3	2.392 (1.157)	2.534 (1.249)	[−0.359; 0.075]	*t*(254) = −1.291, *p* =0.198	*d* = −0.12 [−0.296; 0.056]
4	2.762 (1.114)	2.81 (1.168)	[−0.251; 0.157]	*t*(260) = −0.458, *p* =0.647	*d* = −0.042 [−0.218; 0.134]
5	2.297 (1.126)	2.638 (1.07)	[−0.533; −0.15]	*t*(282) = −3.514, *p* =0.001	*d* = −0.306 [−0.483; −0.13]
6	2.722 (0.927)	2.798 (0.944)	[−0.241; 0.091]	*t*(266) = −0.891, *p* =0.374	*d* = −0.081 [−0.256; 0.095]
7	1.975 (1.105)	2.11 (1.186)	[−0.341; 0.071]	*t*(255) = −1.291, *p* =0.198	*d* = −0.12 [−0.296; 0.056]
8	1.757 (1.072)	2.049 (1.099)	[−0.485; −0.1]	*t*(264) = −2.986, *p* =0.003	*d* = −0.271 [−0.447; −0.095]
9	2.515 (1.134)	2.681 (1.169)	[−0.371; 0.039]	*t*(263) = −1.593, *p* =0.112	*d* = −0.145 [−0.321; 0.031]
10	2.352 (1.062)	2.632 (1.03)	[−0.463; −0.097]	*t*(277) = −3.012, *p* =0.003	*d* = −0.265 [−0.442; −0.089]
Total	2.437 (.662)	2.611 (0.7)	[−0.296; −0.052]	*t*(258) = −2.808, *p* =0.005	*d* = −0.259 [−0.435; −0.083]

#### Convergent Validity

Correlations between CD-RISC-10 and other measures of well-/ill-being are presented in Table 3.

**Table 3:** Correlations (Pearson *r*) between CD-RISC-10 and other measures of well-/ill-being.

	**CES-D (*n* = 689)**	**PA (*n* = 249)**	**NA (*n* = 249)**	**S-Est (*n* = 687)**	**WEMWBS (*n* = 683)**	**Authentic living (*n* = 684)**	**Accepting external influence (*n* = 673)**	**Self-alienation (*n* = 685)**
*CD-RISC-10*	−0.517^***^	0.295^***^	−0.390^***^	0.578^***^	0.602^***^	0.414^***^	−0.305^***^	−0.419^***^

*** *p < 0.001*.

All correlations are significant at level *p* < 0.001, but the strengths of the correlations are different. There were high positive correlations with the *Self-Esteem Scale* and WEMWBS; a high negative correlation with CES-D; a moderate positive correlation with the *authentic living* subscale; moderate negative correlations with *self-alienation* and the NA scale; a weak positive correlation with the PA scale; and a weak negative correlation with *accepting external influence*.

To sum up, these results are in line with previous research and confirm Hypotheses 2a and 2b, which demonstrate good convergent validity of the Russian version of CD-RISC-10.

## Discussion

The current paper is dedicated to the validation of one of the most popular and effective diagnostic tools, CD-RISC-10, in Russia. Each culture needs such an instrument for monitoring the mental health and emotional tension in the population in stable and crisis situations. The availability of such a tool is absolutely necessary for different areas of research, psychological, and educational practice.

The correlational research included some steps typical for the psychometric examination of the new tool—translating, checking reliability and structural and convergent validity, and investigating age trends and sex differences. Both hypotheses about psychometric properties of CD-RISC-10 were confirmed.

As expected and in accordance with previous results (7–9, 13, 15–17, 30, 31, 34–37, 41), we have confirmed the unifactorial structure of the questionnaire. It means that resilience, in accordance with its understanding by Connor and Davidson and later by Campbell and Sills, is a fairly uniform, internally consistent, and cross-culturally invariant personality trait that can really be measured with one simple instrument.

We have also revealed the reliability of the tool (α = 0.85, ω_*h*_ = 0.84), which is the same as in the original short version (α = 0.85, 15). Furthermore, we have revealed clear results regarding convergent validity. In line with She et al. (17), we have received positive connections between CD-RISC-10 and WEMWBS scores, indicating that our adaptation of the scale really measures resilience as an adaptive feature. According to Alarcón et al. (16) and She et al. (17), CD-RISC-10 scores were positively correlated with positive affect and negatively correlated with negative affect. Similar to results received by Alarcón et al. (16), Aloba et al. (14), and She et al. (17), CD-RISC-10 was positively correlated with self-esteem. We have revealed the expected negative connection between resilience and depressive symptoms, which is consistent with the findings of Levey et al. (31), Hébert et al. (8), She et al. (17), and Serrano-Parra et al. (34). And, finally, we found positive connections with the *authentic living* subscale, whereas connections with the *accepting external influence* and *self-alienation* subscales were negative. All these results give evidence for the good divergent validity of the Russian adaptation of CD-RISC-10. To sum up, now we have an instrument to measure resilience in Russia, which widely extends the opportunities for clinical and cross-cultural research in a broad spectrum of psychological fields.

## Conclusion

This paper reports that the correlational validation study results in a valid, reliable, and short Russian version of CD-RISC-10. We replicated typical ways of adapting this tool to other cultures. As expected, the Russian version of CD-RISC-10 maintained its unifactorial structure. In line with the results obtained in other cultures, CD-RISC-10 scores correlated positively with mental well-being indicators and correlated negatively with indicators of ill-being. We can conclude that the aim of our research has been achieved.

Nevertheless, the current study is not free of some limitations; the most important of them might be overcome by adding some more objective behavioral or demographic variables, such as school attendance, financial hardship, history of childhood abuse, loneliness, and household dysfunction (17, 31). Another direction of further research is a more detailed investigation of relationships between resilience and other constructs of well-being. Furthermore, we investigated students only and would like to extend our sample to various ages and social strata. We also plan to investigate people experiencing different stress factors and select objective behavioral indicators to further validate the tool and check its invariance in different samples. Finally, an examination of the divergent validity of CD-RISC-10 is an additional line of future research.

Despite the listed limitations of the current study, the new method can be recommended for psychological research related to stress, challenges, or changing and unpredictable life situations. Also, we can expect that the tool will be widely used by consultants working in psychological services to monitor the resilience of students, clinical patients, and professionals in high-risk occupations—athletes, military personnel, rescuers, etc.—as most of them belong to the youth group.

## Data Availability Statement

The raw data supporting the conclusions of this article will be made available by the authors, without undue reservation.

## Ethics Statement

The studies involving human participants were reviewed and approved by National Research University Higher School of Economics Committee on Interuniversity Surveys and Ethical Assessment of Empirical Research. The patients/participants provided their written informed consent to participate in this study.

## Author Contributions

SN-B developed the main idea of the paper, collected the data, organized the database, wrote the first draft of the manuscript, contributed to the manuscript revision, and read and approved the submitted version. AK contributed to the study's conception and design, performed the statistical analysis, contributed to the manuscript revision, and read and approved the submitted version. KB collected the data, organized the database, provided feedback, and read and approved the submitted version. All authors approved the submitted version of the manuscript.

## Conflict of Interest

The authors declare that the research was conducted in the absence of any commercial or financial relationships that could be construed as a potential conflict of interest.

## Footnotes

^1^For the permission to use the Russian version of CD-RISC-10, please contact Dr. Jonathan Davidson (*mail@cd-risc.com*).
